# General Practitioners' opinions on their practice in mental health and their collaboration with mental health professionals

**DOI:** 10.1186/1471-2296-6-18

**Published:** 2005-05-02

**Authors:** Nadia Younes, Isabelle Gasquet, Pierre Gaudebout, Marie-Pierre Chaillet, Viviane Kovess, Bruno Falissard, Marie-Christine Hardy Bayle

**Affiliations:** 1 Academic Unit of Psychiatry, Centre Hospitalier de Versailles, 177 Rue de Versailles 78157 Le Chesnay Cedex. France; 2 National Institute of Health and Medical Research (INSERM-U669), Hôpital Cochin, Paris, France; 3 Direction of Medical Policy, Assistance Publique – Hôpitaux de Paris, paris, France; 4 MGEN, Mental Health Foundation, Paris, France

## Abstract

**Background:**

Common mental health problems are mainly treated in primary care settings and collaboration with mental health services is needed. Prior to re-organisation of the mental health care offer in a geographical area, a study was organized: 1) to evaluate GPs' opinions on their day-to-day practice with Patients with Mental Health Problems (PMHP) and on relationships with Mental Health Professionals (MHPro); 2) to identify factors associated with perceived need for collaboration with MHPro and with actual collaboration.

**Methods:**

All GPs in the South Yvelines area in France (n = 492) were informed of the implementation of a local mental health program. GPs interested in taking part (n = 180) were invited to complete a satisfaction questionnaire on their practice in the field of Mental Health and to include prospectively all PMHP consultants over an 8-day period (n = 1519). For each PMHP, data was collected on demographic and clinical profile, and on needs (met v. unmet) for collaboration with MHPro.

**Results:**

A majority of GPs rated PMHP as requiring more care (83.4%), more time (92.3%), more frequent consultations (64.0%) and as being more difficult to refer (87.7%) than other patients. A minority of GPs had a satisfactory relationship with private psychiatrists (49.5%), public psychiatrists (35%) and social workers (27.8%). 53.9% had a less satisfactory relationship with MHPro than with other physicians.

Needs for collaboration with a MHPro were more often felt in caring for PMHP who were young, not in employment, with mental health problems lasting for more than one year, with a history of psychiatric hospitalization, and showing reluctance to talk of psychological problems and to consult a MHPro.

Needs for collaboration were more often met among PMHP with past psychiatric consultation or hospitalization and when the patient was not reluctant to consult a MHPro. Where needs were not met, GP would opt for the classic procedure of mental health referral for only 31.3% of their PMHP.

**Conclusion:**

GPs need targeted collaboration with MHPro to support their management of PMHP, whom they are willing to care for without systematic referral to specialists as the major therapeutic option.

## Background

In developed countries, mental health problems, especially anxious and depressive disorders, are frequent and a leading cause of disability [1-4]. Since they are potentially remediable when adequately treated, they represent a major public health challenge [5,6]. A major obstacle to the instatement of adequate care is that when people do seek help, generally from their General Practitioner (GP), most of these problems are not recognized or not appropriately treated [4,7-9]. GPs have thus received special attention to improve mental health care because of their unique position [10]. Educational interventions have been proposed but have shown some limitations: temporary effect, no improvement in recognition of depression nor in patient recovery. They seem effective only when accompanied by organizational interventions [10-13]. Organizational interventions, based on the interaction between primary and secondary care, have been developed in several countries through local initiatives or national mental health reforms for improving depression care: in US [13,14], in UK [15], in Australia [16,17], in Canada [18,19]. They focus on the key role of GPs and on different forms of collaboration with mental health professionals (education, communication, on-site collaboration, collaborative care, stepped collaborative care, quality improvement, case management...). In France, collaboration is also encouraged by national government policies ("plan santé mentale" 2001 and 2005–2008). However collaboration of this sort requires pragmatic definition in clinical practice: for which patients with mental health problems (PMHP) do GPs need assistance from Mental Health Professionals (MHPro)? What sort of assistance? Why has this assistance not been organized up till now, i.e. what are the barriers to collaboration? Defining these issues is important before the development of quality improvement programs, considering some disappointing instances of collaboration between GPs and psychiatrists, where GPs have made limited use of opportunities for collaborative care with psychiatrists in spite of GP-reported perceived needs [20].

To design effective quality improvement programs based on targeted strategies among professionals and adapted to professional needs in the pilot area of South Yvelines, a survey was organized to gather information on some of these questions, with two objectives.

First, to evaluate satisfaction with mental health practice, exploring GPs' opinions on their patients with mental health problems (PMHP) and on relationships with Mental Health Professionals (MHPro).

Second, to measure factors associated with GPs' needs for collaboration with MHPro, with collaboration actually occurring, and with instances where needs are not met.

## Methods

### Population

All the 492 GPs of the area of "South Yvelines" (600 000 inhabitants) were approached by post in spring 2000 to recruit for the survey with a postage-paid reply envelope if they agreed to take part in this local area mental health program.

GPs were asked to include prospectively over an 8-day period all consulting patients over 15 years old "for whom a Mental Health Problem was the main current problem".

### Data collected

GPs completed two questionnaires requiring approximately 30 minutes to complete:

First a questionnaire on their overall practice in the field of mental health, including data on their opinions on their PMHP compared to other patients, and on relationships with MHPro compared to other physicians.

A second questionnaire was completed for each PMHP included. Data was collected on demographic profile, clinical status, care provision and needs for collaboration with MHPro (met or unmet). To be feasible in daily practice, diagnoses were established using a classification developed by a working group of GPs and psychiatrists, secondarily translated into CIM-10 main diagnostic groups by 2 physicians independent from the study (a psychiatrist and a GP).

### Statistical analysis

Descriptive and comparative analyses were performed with SAS 8.2. Three groups of PMHP were considered: no need for collaboration with MHPro expressed by the GP ("No need"); need for collaboration with MHPro but no actual collaboration ("Need Unmet") and need for collaboration with MHPro and actual collaboration ("Need Met"). Factors leading to a need for collaboration ("Need") and to actual collaboration ("Need met") were determined using two multivariate logistic regressions. The patients' demographic and clinical variables were entered into the regressions where chi-square tests (for categorical variables) and ANOVA tests (for continuous variables) produced a 5% level of significance. The "need" multivariate logistic regression, obtaining a non-significant result on the Hosmer and Lemeshow Goodness-of-Fit Test (p = 0.95), concerned 1007 patients. The "need met" multivariate logistic regression also producing a non-significant result on the Hosmer and Lemeshow Goodness-of-Fit Test (p = 0.87), concerned 532 patients.

## Results

### Characteristics of participating GPs and patients enrolled

One hundred and eighty GPs volunteered to participate to the mental health program (36.6% of local area GPs). They were predominantly male (69.4%), experienced providers (66.1% had been working for more than 10 years) and most were exclusively in private practice (79.4%). Compared to local area GPs, they were younger (p = 0.05) but did not differ for gender (Table 1).

**Table 1:** Characteristics of GPs responding to the survey (N = 180)

	Respondents N = 182	Yvelines GPs N = 492	P
Male (%)	69.4	78.4	ns (0.3)

Age in years (%)			
25 to 34	9.4	7.7	
35 to 44	40.6	45.1	
45 to 54	43.9	37.0	0.05
55 to 64	5.6	8.7	
65 and over	0.6	1.6	

Time in current practice (%)			
< 5 years	15.0	na	
5 to 10	18.9	na	
> 10	66.1	na	

Type of practice (%)			
Private practice exclusively	79.4	na	
Private and public practice	20.6	na	

Mean working hours per week (sd)	51.5(15)	na	

na Data not available

The GPs enrolled 1519 MHP patients, representing 15.0 % of the overall number of consultations. Each participating GP saw 8 MHP patients on average (range 0–35). A majority of MHP patients were female (68.2%), mean age was 46.9 years (sd = 15.9). 61.4% had a current professional activity, 25.5% were living alone and 13.7% had a national disability allowance. The most frequent diagnoses were anxious and depressive disorders (33.7% and 31.3%). The disorders had lasted on average for 6.7 years (sd = 8.1). 18.3% of patients had a history of psychiatric hospitalization, 51% a history of care by psychiatrists. 71.6% had been managed by GPs for more than 2 years. Consultations lasted on average 23.2 minutes (sd = 8.9). According to the GPs, for 70.8% it was easy to talk about "psychological problems" but it was less easy to talk about a psychiatric consultation (proving easy for only 43.4%).

### GPs' opinions on Patients with Mental Health Problems and on relationships with Mental Health Professionals

Four GPs out of five considered that patients with MHP have more expectations regarding care (83.4%), require more consultation time (92.3%) and are more difficult to refer to a specialist (87.7%) than other patients. A majority of GPs (64.2%) regretted having so many patients with MHP. 46.6% considered that PMHP expectations in terms of medical results are greater than among other patients. Few GPs complained about non-punctuality or unreliability of PMHP with regard to appointments (14.4%) (Table 2).

**Table 2:** GPs' opinion on their Patients with Mental Health Problems compared to their other patients (N = 182)

%	Fully agree	Rather agree	Rather disagree	Completely disagree	No opinion
Have more expectations for care	30.0	53.4	10.5	1.1	5,0
Have more expectations for results	9.4	37.2	45.6	2.8	5,0
Expect more frequent consultations	22.2	42.8	26.1	3.3	5.6
Require more time	58.9	33.4	4.4	1.1	2.2
Are more difficult to refer	53.3	34.4	6.7	3.9	1.7
Are less punctual/reliable on appointments	7.7	6.7	27.8	51.7	6.1

While 78.4% of GPs were 'very' or 'mostly' satisfied with their relationships with other GPs, only 49.5% rated the same level of satisfaction for relationships with private psychiatrists, 35.0% for public psychiatrists and 27.8% for social workers. None of the GPs was 'very' satisfied with the information given by mental health professionals, and only 23.9% were mostly satisfied (Table 3).

**Table 3:** GPs' satisfaction of quality of exchanges with Health Professionals (N = 182)

%	Very satisfied	Fairly Satisfied	Fairly Unsatisfied	Very unsatisfied	No opinion
Relationship with					
...private psychiatrists	6.7	42.8	36.1	11.7	2.8
...public psychiatrists	3.5	31.5	21.8	8.1	35.1
...with social workers	1.1	26.7	37.8	10.6	23.8
...other primary physicians	17.8	60.6	16.7	1.7	3.3
...health professionals in general	9.5	73.9	14.4	1.7	0.6
Information received from mental health professionals in case of collaboration for a patient	0.0	23.9	42.8	28.9	4.4
					
	Much better	Better	Same	Worse	Much worse

Relationship with mental health professionals in comparison with other health colleagues	0.6	4.5	41.0	40.4	13.5

### Factors associated with GPs' needs for collaboration with Mental Health Professionals and with these needs being met

GPs felt a need for collaboration with a MHPro for 43.3% of their MHP patients. Within this group only 35.3% felt that their need was met (15.3% of the overall PMHP group).

Where needs were not met, for 64.1% of their patients GPs do not know what type of collaboration to seek, and for 31.3% they considered there was a need for care by MHPro, and for occasional advice for 4.6%. They would like to be able to refer mainly because they lack confidence with this type of care (48.3 %) but also because it requires too much time (17.8%). 70.5% cited a psychiatrist as the desired collaborator, and 22.7% a psychologist.

The need for collaboration with a MHPro (whether met or unmet) was more often felt by GPs for PMHP who were young (p < .0001), not in employment (p = .002), with mental health problems lasting for more than 1 year (p = .003) and past psychiatric hospitalization (p < .0001). GPs' needs for collaboration were more frequent when the patient was reluctant to consult a MHPro. Further to this, GPs seem to be rather more comfortable with patients suffering from anxiety than with other diagnoses. Finally, where a need for collaboration was felt, consultations were shorter (Table 4, ' [see Additional file 1]').

The need for collaboration was more often met in case of past psychiatric consultation (p = .0002) or hospitalization (p = .0004) and when the patient showed no reluctance to consult a MHPro (Table 4, ' [see Additional file 1]').

The more emphasis GPs put on collaboration, the more positive they evaluated their relationships with mental health professionals to be. 57.5% considered relationships with MHPro as less satisfactory than those with other health professionals when no need for collaboration was felt, 55.9% in case of unmet need and 48.1% when need was met (p = 0.004).

## Discussion

### Limitations

More than one third of GPs contacted volunteered for the local area mental health program and participated in the study. Results may reflect a particular population of GPs, younger than the average and probably already more involved in mental health care in their ordinary practice than non-respondents (who were however not contacted). It is likely that mental health actions targeting GPs can reach only a certain proportion. It has indeed been shown that the willingness to collaborate is greater among physicians under the age of 50 [21].

Caution is also required in the interpretation of this study on account of a second limitation. This resides in the fact that the results are based on GPs' reports on patients that they identified as PMHP. This use of assessment by the GPs could involve a recruitment bias, with a selection of the most severe patients. Indeed, external audits among general practice attendees have shown high unmet needs of mental health treatments but also PMHP as having less severe, less chronic and more readily treatable disorders [22-25]. The study option was to approach GPs' day to day practice with such patients and their subjective perceptions. The focus is on their attitudes towards patients they identify as PMHP and their attitudes towards the relevant specialist services, the aim being to adapt the mental health program to these particular attitudes.

### GPs' opinions on their Patients with Mental Health Problems

In the study, PMHP identified by primary care respondents presented mainly anxious and depressive disorders. GPs have rather negative attitudes towards them. Previous papers have noted that complicated depressive symptoms are frequently encountered in primary care [26-28] and PMHP are time-consuming and require particular skills [29]. But as shown in our study, managing PMHP is a key part of a GP's job, and a part they are willing to take on if sufficient support and expertise are available.

### GPs' collaboration with Mental Health Professionals

In the survey, GPs' needs for collaboration with MHPro have been reported to apply to half of their MHP patients. No publication on this point was found in the literature. Other studies conducted in ordinary practice have been focused on actual referrals from GPs to MHPro, or on actual utilization of mental health specialists, without reporting on GPs' perceived needs as is the case here [26,28,30,31]. Referral percentages have been estimated to be between 4 to 23% of primary care patients, and utilization of mental health specialists at 38% of depressed patients [26,30,31]. GPs' perceived needs for collaboration with MHPro are greater than needs for referral, probably because most patients are reluctant to consult a mental health professional [32].

This study sheds new light on factors related to GPs' collaboration with MHPro. According to a previous study on primary care patients with depressive symptoms, the best predictors of referral and utilization of mental health specialists were: more severe depressive symptoms, more long-standing problems (more than 1 year), prior visits to a mental health specialist, more years of education, being in the younger age groups, and being female [31]. The influence of the "psychiatric label" has been shown [33]. The present results on perceived needs for collaboration with MHPro may well apply to all mental pathologies encountered in primary care. Some of the above variables already reported to be related to perceived need (young age, prior mental health care) have been confirmed in the present study, and in addition this work has pinpointed the variable of not being in employment (which could correlate with disease severity). As has already been shown, GPs view patient-centered barriers as the most influential barrier to collaboration, more so than physician-centered barriers or system barriers [26,28-30,34,35]. But these patient-centered barriers could be associated with physician centered barriers, given GPs' dissatisfaction with relationships with mental health professionals. The dissatisfaction is greater than with other health professionals, and dissatisfaction is known to be associated with less frequent use of mental health services [30]. It is noteworthy that when needs are not met, only a third of GPs would opt for a referral to MHPro, suggesting that it is not a major therapeutic option for GPs. The classic pattern of referral to specialists as the major therapeutic option is often not relevant since it does not readily occur in day-to-day practice. The solution could be to develop other forms of collaboration between GPs and mental health professionals. Many MHP patients could be managed entirely by their GPs or treated in primary care if sufficient expertise is available (prompt psychiatric consultation, collaborative care) without actual referral [10,13,25]. To reinforce this notion, our results have shown that when there is actual collaboration, GPs' negative opinions on relationships with mental health professionals are less marked.

## Conclusion

GPs are a key factor in the care of the commoner mental health problems. They are willing to care for this type of patient if they have more support for this job than they do at present. There is a need for collaboration, not in the form of the classic referral to specialists as the major therapeutic option, but in the form of emphasis on collaborative relationships with mental health specialists, to improve quality of the care provided in commoner mental health disorders[36]. Results from this survey have been integrated into the "South Yvelines Mental Health Network" created in June 2001, by promoting this type of collaborative relationships in the area. Further evaluations are underway.

## List of abbreviations

GPs (General Practitioners). MHP (Mental health problems). PMHP (Patients with Mental Health Problems). MHPro (Mental Health Professionals).

## Competing interests

The author(s) declare that they have no competing interests.

## Authors' contributions

Study concept and design : Gasquet, Kovess, Hardy-Bayle. Acquisition of data, study supervision : Chaillet. Analysis and interpretation: Younès, Gasquet. Drafting of the manuscript : Younès. Statistical expertise : Younès, Gaudebout, Falissard. Critical revision : Gasquet, Younès, Falissard, Kovess, Hardy-Bayle

## Pre-publication history

The pre-publication history for this paper can be accessed here:



## Supplementary Material

Additional File 1 Table 4 : Primary care patients' factors associated with Needs, Needs met and Needs unmet for collaboration with Mental Health Professionals. Univariate analysis and logistic regressions for demographic profile, clinical profile, modality of primary care, past psychiatric care, patient's attitude towards psychological problems. Click here for file
