# Supplementary material for: General Practitioners' opinions on their practice in mental health and their collaboration with mental health professionals
Source: BMC Fam Pract. 2005 May 2;6:18. doi: 10.1186/1471-2296-6-18 (PMC1131897; doi:10.1186/1471-2296-6-18)
Supplement: Additional File 1 — Table 4 : Primary care patients' factors associated with Needs, Needs met and Needs unmet for collaboration with Mental Health Professionals. Univariate analysis and logistic regressions for demographic profile, clinical profile, modality of primary care, past psychiatric care, patient's attitude towards psychological problems. [file 1471-2296-6-18-S1.doc]

Table 4: Primary care patients’ factors associated with Needs, Needs met and Needs unmet for collaboration with Mental Health Professionals

|  |  | **Need** | |  | **Univariate Analysis** | | **Logistic Regression** | |
| --- | --- | --- | --- | --- | --- | --- | --- | --- |
|  | **No need (1)**  **n=591**  **56.8%** | **Need Unmet (2)**  **n=291**  **27.9%** | **Need Met (3)**  **n=159**  **15.3%** |  | **(1) v. (2+3)**  **p** | **(2) v. (3)**  **p** | **(1) v. (2+3)**  **0Ra§ [CI95%]** | **(2) v. (3)**  **0Ra§§[CI95%]** |
| Demographic profile |  |  |  |  |  |  |  |  |
| Mean age in years (sd) | 50.5 (16.2) | 47.1 (15.3) | 45.5 (15.2) |  | ******* | ns | **0.97 [0.96-0.98]***** | - |
| Gender: male (%) | 30.3 | 33.3 | 29.4 |  | ns | ns | - | - |
| Current professional activity (%) | 62.8 | 57.8 | 55.8 |  | ***** | ns | **1.6 [1.15-2.23]*** | - |
| Living alone (%) | 27.4 | 26.4 | 24.6 |  | ns | ns | - | - |
| Clinical profile(%) |  |  |  |  |  |  |  |  |
| Diagnoses |  |  |  |  |  |  |  |  |
| Anxious disorders | 30.5 | 23.0 | 19.5 |  | ****** | ns | **0.5 [0.37-0.78]** ** | - |
| Depressive disorders | 27.3 | 28.2 | 29.0 |  | ns | ns | - | - |
| Bipolar disorder | 0.6 | 0.0 | 4.4 |  | ***** | ****** | - | - |
| Substance use | 4.1 | 9.6 | 6.3 |  | ****** | ns | 1.02 [0.53-1.96] | - |
| Delusion | 6.3 | 4.1 | 1.8 |  | ***** | ns | 1.3 [0.51-3.12] | - |
| Personality disorders | 0.0 | 0.0 | 0.0 |  | - | - | - | - |
| Eating disorders | 1.0 | 1.4 | 2.2 |  | ns | ns | - | - |
| Behavior disorders | 2.6 | 3.4 | 2.9 |  | ns | ns | - | - |
| Family troubles | 12.4 | 7.9 | 7.0 |  | ***** | ns | 0.6 [0.38-1.05] | - |
| Duration of the MHP (%) |  |  |  |  |  |  |  | - |
| > 1 year | 33.5 | 27.6 | 19.3 |  | ******* | ns | 1 |  |
| 1 - 3 years | 16.9 | 17.2 | 16.3 |  |  |  | **1.6 [1.04-2.55]*** |  |
| > 3 years | 49.6 | 55.2 | 64.7 |  |  |  | **1.5 [1.03- 2.18]*** |  |
| Modality of primary care |  |  |  |  |  |  |  |  |
| Mean duration of care in years (sd) |  |  |  |  |  |  |  |  |
| Less than 1 year | 14.8 | 15.7 | 13.6 |  | ns | ns | - | - |
| 1 to 2 years | 11.5 | 14.7 | 13.2 |  |  |  |  |  |
| More than 2 years | 73.6 | 69.6 | 73.3 |  |  |  |  |  |
| Mean duration of consultation in minutes (sd) | 24.1 (8.7) | 23.8 (8.8) | 22.3 (8.5) |  | ***** | ns | **1.03 [1.01-1.05]*** | - |
| Past psychiatric care (%) |  |  |  |  |  |  |  |  |
| Psychiatrist consultation |  |  |  |  |  |  |  |  |
| No | 21.8 | 45.4 | 53.1 |  | ******* | ******* | 1 | 1 |
| Yes | 76.4 | 47.4 | 42.8 |  |  |  | **1.5 [1.03-2.06] *** | **2.4 [1.52-3.85]**** |
| Unknown | 1.8 | 7.2 | 4.0 |  |  |  | 1.8 [0.80-3.91] | 0.7 [0.19-2.28] |
| Psychiatric hospitalization |  |  |  |  |  |  |  |  |
| No | 54.2 | 72.3 | 86.4 |  | ******* | ******* | 1 | 1 |
| Yes | 41.7 | 19.0 | 10.4 |  |  |  | **2.8 [1.85-4.33]***** | **5.4 [2.04-14.35]**** |
| Unknown | 4.1 | 8.6 | 3.2 |  |  |  | **1.6 [0.78-3.87]*** | **2.7 [1.05-6.82] *** |
| Patient’s attitude towards psychological problems (%) | | |  |  |  |  |  |  |
| The patient admits psychological problem | | |  |  |  |  |  |  |
| No (or very reluctantly) | 19.8 | 32.7 | 21.8 |  | ***** | ****** | 1 | 1 |
| Yes (or fairly readily) | 79.4 | 67.0 | 77.9 |  |  |  | **0.6 [0.38-0.82]**** | 0.6 [0.3-1.02] |
| Unknown | 0.7 | 0.3 | 0.2 |  |  |  | 1.6 [0.15-16.78] | 3.6 [0.25-49.96] |
| Reluctance to consult a MHPro (%) |  |  |  |  |  |  |  |  |
| Yes | 20.2 | 65.3 | 36.1 |  | ******* | ******* | 1 | 1 |
| No | 75.0 | 33.7 | 40.9 |  |  |  | 1.2 [0.86-1.68] | **8.8 [5.24-14.80]** ******* |
| Not discussed with the patient | 4.8 | 1.0 | 23.1 |  |  |  | **0.1 [0.06- 0.21]***** | **13.4 [3.35-53.89]** ****** |

- : non introduced in logistic regression (variable non significant in univariate analysis or n too small (bipolar disorder)) ns : non significant, * p=0.05, ** p=0.01, *** p= 0,001.

§ Logistic regression (n= 1007). Variables entered in the model : age, mean duration of consultation, current professional activity, past psychiatrist consultation, anxious disorders diagnosis, substance use diagnosis,

delusion diagnosis, family troubles diagnosis, past psychiatrist hospitalization, easiness to talk of psychological problems with patient, patient’ reluctance to consult a MHPro

§§ Logistic regression (n= 532). Variables entered in the model : past psychiatrist consultation, past psychiatrist hospitalization, easiness to talk of psychological problems with patient, patient’ reluctance to consult a MHPro
